# Performance of the LumiraDx Microfluidic Immunofluorescence Point-of-Care SARS-CoV-2 Antigen Test in Asymptomatic Adults and Children

**DOI:** 10.1093/ajcp/aqab173

**Published:** 2021-10-20

**Authors:** Paul Drain, Raed Sulaiman, Melanie Hoppers, Nigel M Lindner, Vicki Lawson, Jayne E Ellis

**Affiliations:** Department of Global Health and the Department of Medicine, University of Washington, Seattle, WA, USA; Avera Research Institute, Sioux Falls, SD, USA; Clinical Research Solutions, Jackson, TN, USA; LumiraDx UK, London, UK; LumiraDx UK, London, UK; LumiraDx UK, London, UK

**Keywords:** COVID-19, LumiraDx antigen test, RT-PCR, SARS-CoV-2, Sensitivity, Asymptomatic

## Abstract

**Objectives:**

The LumiraDx SARS-CoV-2 Ag Test has previously been shown to accurately detect severe acute respiratory syndrome coronavirus 2 (SARS-CoV-2) in individuals symptomatic for coronavirus disease 2019 (COVID-19). This evaluation investigated the LumiraDx SARS-CoV-2 Ag Test as an aid in the diagnosis of SARS-CoV-2 infection in asymptomatic adults and children.

**Methods:**

Asymptomatic individuals at high risk of COVID-19 infection were recruited in 5 point-of-care (POC) settings. Two paired anterior nasal swabs were collected from each participant, tested by using the LumiraDx SARS-CoV-2 Ag Test at the POC, and compared with results from reverse transcription–polymerase chain reaction (RT-PCR) assays (cobas 6800 [Roche Diagnostics] or TaqPath [Thermo Fisher Scientific]). We calculated positive percent agreement (PPA) and negative percent agreement (NPA), then stratified results on the basis of RT-PCR reference platform and cycle threshold.

**Results:**

Of the 222 included study participants confirmed to be symptom-free for at least 2 weeks before testing, the PPA was 82.1% (95% confidence interval [CI], 64.4%-92.1%). The LumiraDx SARS-CoV-2 Ag Test correctly identified 95.8% (95% CI, 79.8%-99.3%) of the samples confirmed positive in fewer than 33 RT-PCR cycles and 100% (95% CI, 85.1%-100%) in fewer than 30 RT-PCR cycles while maintaining 100% NPA.

**Conclusions:**

This rapid, high-sensitivity test can be used to screen asymptomatic patients for acute SARS-CoV-2 infection in clinic- and community-based settings.

**KEY POINTS**

- To date, point-of-care severe acute respiratory syndrome coronavirus 2 (SARS-CoV-2) antigen tests have shown poor sensitivity in asymptomatic people.
- The LumiraDx SARS-CoV-2 Antigen (Ag) Test evaluation in asymptomatic patients demonstrated 82.1% positive percent agreement and 100% negative percent agreement compared with reverse transcription–polymerase chain reaction.
- This test offers rapid, high-sensitive screening of asymptomatic patients for acute SARS-CoV-2 infection in clinic- and community-based settings.

## INTRODUCTION

Only months after the identification of a novel virus—severe acute respiratory syndrome coronavirus 2 (SARS-CoV-2)—and its associated disease—coronavirus disease 2019 (COVID-19)—tens of thousands of cases were reported in more than 100 countries.^1,2^ The World Health Organization declared the SARS-CoV-2 outbreak a public health emergency of international concern in January 2020,^3^ followed by the declaration of a pandemic on March 11, 2020.^4^ One of the first major problems encountered in the evaluation and monitoring of the pandemic was the lack of diagnostic resources for COVID-19. In response, the Secretary of the US Department of Health and Human Services issued an Emergency Use Authorization (EUA) declaration in early February 2020 for the diagnosis of SARS-CoV-2.^5^

Accurate diagnosis is essential for identifying and managing SARS-CoV-2–infected patients and for the implementation of effective infection-control measures. Currently, the most sensitive testing method for the presence of SARS-CoV-2 is reverse transcription–polymerase chain reaction (RT-PCR) using nasopharyngeal swabs. This method has drawbacks for community-based asymptomatic screening, however, such as the requirement for laboratory testing and subsequent delays in reporting results to individuals, who may not isolate themselves until their result has been confirmed. Currently, high-sensitivity, rapid molecular tests for the identification of SARS-CoV-2 are available, but these tests are often difficult to deploy in community settings, such as drive-in hubs, schools, and workplaces. This difficulty is mainly because test cartridges require refrigeration, are not easily portable, can be difficult to connect directly to surveillance monitoring, and can be expensive to operate. Therefore, rapid molecular tests are not available for widespread use.^6^ Affordable, easy-to-use, rapid (results in 10-30 minutes), and accurate diagnostic tests that can be used in local clinics at the point of care (POC) can help alleviate the burden that the COVID-19 pandemic has exerted on health care systems.^7^ The US government called for the development of rapid POC SARS-CoV-2 antigen tests, which as of September 21, 2021, led to the US Food and Drug Administration (FDA) EUA of 34 rapid diagnostic tests that can be performed at the POC in any health care setting.^8,9^ Variations in sensitivity and specificity have been reported, however, and many of the available SARS-CoV-2 rapid antigen tests have now been demonstrated to have a sensitivity below 80%, which means that there is a high chance that infected individuals could receive a false-negative test result.^10-15^ When individuals receive a false-negative test result, these infected people, who may be asymptomatic, are not quarantined and thus contribute to the transmission of SARS-CoV-2.^16^ This effect highlights the importance of evaluation studies to assess the sensitivity and specificity of each diagnostic test available on the market; these analyses can facilitate informed decision-making when selecting a test to use.

SARS-CoV-2 rapid antigen tests have been shown to have lower diagnostic sensitivity for samples obtained from asymptomatic people compared with symptomatic people.^13,15,17,18^ Brümmer and colleagues^18^ reported substantially lower accuracy for antigen tests in asymptomatic people (52.5%) compared with symptomatic people (76.7%), and the Abbott BinaxNOW rapid antigen test was reported to have a sensitivity of 35.8% in asymptomatic people compared with 64.2% in symptomatic people.^15^ Because asymptomatic people have no means of identifying how long they have been infected or when they became infected because of the lack of symptoms, data review suggests that these tests have a wider RT-PCR cycle threshold (Ct) range and probably include people who are carrying remnant SARS-CoV-2 RNA rather than viable virus.^18^ Brümmer and colleagues^18^ estimated, based on 61 antigen tests, a range of Ct values from 20.5 to 27 for symptomatic patients and from 27.2 to 30.5 for asymptomatic atients. The lower sensitivity of lateral flow antigen tests indicates that these tests are better suited to testing symptomatic people in the early stages of infection.^18^ To date, no POC SARS-CoV-2 rapid antigen test for professional use has received FDA EUA for use in asymptomatic people without required serial testing over 48 to 72 hours.^9^ When an asymptomatic person in a congregate living setting has a high likelihood of SARS-CoV-2 infection and tests negative using a SARS-CoV-2 rapid antigen tests, a confirmatory test within 48 hours is recommended.^19^

The LumiraDx SARS-CoV-2 Ag Test is a microfluidic immunofluorescence POC assay for direct and qualitative detection of SARS-CoV-2–specific nucleocapsid protein antigen in nasal and nasopharyngeal swab specimens.^20,21^ Recently, the LumiraDx SARS-CoV-2 Ag Test was evaluated for diagnosing acute COVID-19 in adults and children and was determined to detect 97.6% of COVID-19 infections compared with reference RT-PCR testing in symptomatic patients.^20^ The test received initial FDA EUA for testing of symptomatic individuals suspected of having COVID-19 within 12 days of symptom onset and is designed to deliver test results in 12 minutes.^22^ The current prospective study aimed to evaluate the performance of the LumiraDx SARS-CoV-2 Ag Test, which uses microfluidic technology, in asymptomatic adults and children.

## MATERIALS AND METHODS

### Study Participants

Adult (aged ≥18 years) and pediatric (aged ≤17 years) participants were consecutively recruited between November 13, 2020, and March 25, 2021, from 5 sites across the United States (Avera Research Institute, Sioux Falls, SD; Eclipse Clinical Research, Jackson, TN; CVS Health, Atlanta, GA; Village Health Partners, Plano, TX; and MRN Diagnostics, Franklin, MA) in this prospective study (ASPIRE study; NCT04557046 and MRN Diagnostics Protocol). Eligible participants for this evaluation had been in contact with someone who had tested positive for SARS-CoV-2 or had been confirmed positive themselves within 48 hours before recruitment and were asymptomatic at the time of testing. Asymptomatic participants were defined as those not experiencing current COVID-19 symptoms and without having experienced COVID-19 symptoms within 2 weeks of testing. COVID-19 symptoms included fever, chills, shortness of breath, vomiting, difficulty breathing, new loss of taste or smell, diarrhea, cough, sore throat, headache, nausea, body ache, and runny nose.

### Study Design

A total of 2 anterior nasal swabs were consecutively collected from each participant by inserting a swab in each nostril, and then exchanging the swab into the second nostril to ensure that a sample from each nostril was collected on each swab and to minimize bias between swabs. One swab was processed for sample collection using the LumiraDx extraction buffer and further processed and tested according to the LumiraDx SARS-CoV-2 Ag Test product insert.^22^ The second (reference) swab was placed in 3 mL of viral transport media (Universal Viral Transport Medium, BD Life Sciences) and tested by RT-PCR at 1 of 2 reference laboratories: TriCore Reference Laboratories (Albuquerque, NM) using the cobas 6800 SARS-CoV-2 Test^23^ (Roche Diagnostics) or TruGenX (Lyndhurst, NJ) using the TaqPath COVID-19 Combo Kit^24^ (Thermo Fisher Scientific) with a KingFisher Flex System (Thermo Fisher Scientific). The samples were transported on ice with a ColdMark tracker and tested within 48 hours of collection per the manufacturer’s instructions. The operators at the POC test sites were trained in how to prepare and transport the reference test samples to the reference laboratories. Intended-use operators at the POC were blinded to the RT-PCR test result but not symptomatic status. Operators of reference RT-PCR platform were blinded to the result of the LumiraDx SARS-CoV-2 Ag Test.

Ethics approval for the ASPIRE study (NCT04557046) was obtained from WCG IRB. Samples obtained from MRN Diagnostics were collected under their own approved ethics guidelines. Written informed consent was obtained from all participants before enrollment. In addition, study protocols complied with the Declaration of Helsinki (2013). All participants were able to donate samples without compromising their current health status. At the end of the study, we performed a retrospective follow-up to confirm that participants were symptom-free 2 weeks before recruitment and testing. This follow-up was conducted under the approved protocols of WCG IRB.

### Statistical Analysis

This research study was set up in compliance with the FDA Antigen Template for Test Developers, which requires testing a minimum of 30 positive samples and 30 negative samples (confirmed by reference RT-PCR).^25^ For an asymptomatic test, an additional 20 positive asymptomatic participants were required under an existing EUA. Only samples for which both RT-PCR and LumiraDx SARS-CoV-2 Ag Test results were available were included in the analysis. Prespecified statistical analysis to determine the positive percent agreement (PPA) and negative percent agreement (NPA) of the LumiraDx SARS-CoV-2 Ag Test and their associated 2-sided Wilson Score 95% confidence intervals (CIs) was performed using Microsoft Excel, version 16.0.12527.20880. The PPA of the LumiraDx SARS-CoV-2 Ag Test in asymptomatic participants was assessed against acceptance criteria of demonstrating a minimum PPA of 80% or higher. To establish the distribution of the RT-PCR Ct values of the cobas 6800 SARS-CoV-2 Test and TaqPath COVID-19 Combo Kit for asymptomatic participants for whom the Ct values were known, analysis was performed on the Ct subsets of fewer than 33, fewer than 30, and fewer than 25 cycles.

## RESULTS

### Study Population

**FIGURE 1:**
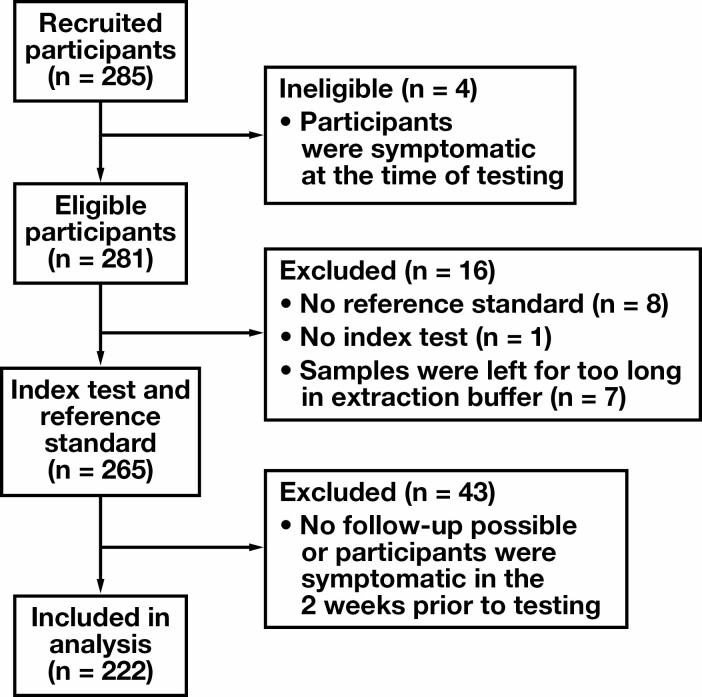
Participant flow diagram.

Of the 285 participants recruited, 222 participants met the inclusion criteria and were included in the data analysis FIGURE 1. The mean (standard deviation) age of included participants was 38.7 (17.3) years (range, 0-88 years), and 63.1% of participants were female TABLE 1. Using an EUA-authorized RT-PCR assay, 28 participants tested positive for the presence of SARS-CoV-2 RNA, and 194 tested negative, giving an overall estimated COVID-19 prevalence of 12.6% in this population TABLE 1. No adverse events following sample collection were reported, and no alternative diagnoses were sought because participants were asymptomatic at the time of testing.

**TABLE 1:** Demographics of the Study Cohort (n = 222)

Demographic	Value
Age, mean (SD), y	38.7 (17.3)
Age, No. (%), y	
≤5	0 (0.0)
6-17	22 (9.9)
18-59	172 (77.5)
≥60	28 (12.6)
Female, No. (%)	140 (63.1)
POC and laboratory testing, No. (%)	
Positive LumiraDx SARS-CoV-2 Ag Test	23 (10.4)
Positive RT-PCR of SARS-CoV-2	28 (12.6)

POC, point of care; RT-PCR, reverse transcription–polymerase chain reaction; SD, standard deviation; SARS-CoV-2, severe acute respiratory syndrome coronavirus 2.

### SARS-CoV-2 Antigen Assay Clinical Validation

Of the 28 samples that tested positive for SARS-CoV-2 RNA by the reference RT-PCR test, the LumiraDx SARS-CoV-2 Ag Test reported 23 positive and 5 negative samples. All RT-PCR SARS-CoV-2–negative samples were confirmed negative by the LumiraDx SARS-CoV-2 Ag Test TABLE 1. The prevalence of positive asymptomatic participants identified by the LumiraDx SARS CoV-2 Ag Test was higher (17.9%) in the older (≥60 years) participant group compared with the younger (<60 years; 9.3%) group TABLE 2. Analysis of the samples obtained from asymptomatic participants indicated a PPA of 82.1% (95% CI, 64.4%-92.1%) and an NPA of 100% (95% CI, 98.1%-100%) for the LumiraDx SARS-CoV-2 Ag Test compared with the reference RT-PCR systems TABLE 2.

**TABLE 2:** Diagnostic Accuracy of the LumiraDx SARS-CoV-2 Ag Test Compared With Reverse Transcription–Polymerase Chain Reaction Assays for Clinical Testing (n = 222)

	TP/(TP+FN), No.	PPA (95% CI), %	TN/(TN+FP), No.	NPA (95% CI), %	PPV (95% CI), %	NPV (95% CI), %	LR+	LR–
Total cohort	23/28	82.1 (64.4-92.1)	194/194	100 (98.1-100)	100 (85.7-100)	97.5 (94.6-98.9)	Inf	0.179
Sex								
F	13/15	86.7 (62.1-96.3)	125/125	100 (97.0-100)	100 (77.2-100)	98.4 (94.4-99.6)	Inf	0.133
M	10/13	76.9 (49.7-91.8)	69/69	100 (94.7-100)	100 (72.2-100)	95.8 (88.5-98.6)	Inf	0.231
Age, y								
<60	18/23	78.3 (58.1-90.3)	171/171	100 (97.8-100)	100 (82.4-100)	97.2 (93.5-98.8)	Inf	0.217
≥60	5/5	100 (56.6-100)	23/23	100 (85.7-100)	100 (56.6-100)	100 (85.7-100)	Inf	0.000

CI, confidence interval; FN, false-negative; FP, false-positive; Inf, infinite; LR, likelihood ratio; NPA, negative percent agreement; NPV, negative predictive value; PPA, positive percent agreement; PPV, positive predictive value; TN, true-negative; TP, true-positive.

**FIGURE 2:**
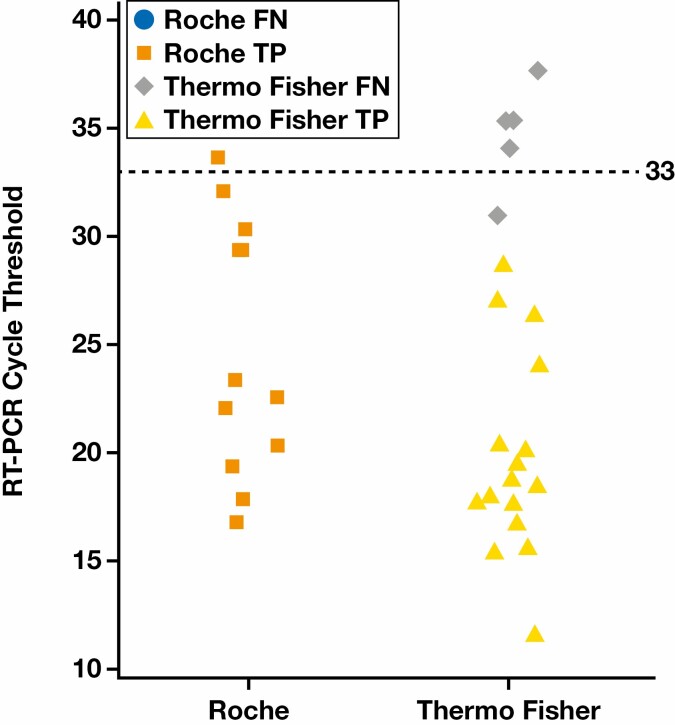
Reverse transcription–polymerase chain reaction (RT-PCR) cycle thresholds for the cobas 6800 SARS-CoV-2 Test (Roche Diagnostics) and TaqPath COVID-19 Combo Kit (Thermo Fisher Scientific). COVID-19, coronavirus disease 2019; FN, false-negative; SARS-CoV-2, severe acute respiratory syndrome coronavirus 2; TP, true-positive.

The agreement between the reference RT-PCR tests and the LumiraDx SARS-CoV-2 Ag Test was highest for samples with lower Ct values, with a PPA of 95.8% (95% CI, 79.8%-99.3%) for samples detected in fewer than 33 cycles (n = 24) TABLE 3. There was no significant difference between the PPA of the LumiraDx SARS-CoV-2 Ag Test obtained from female participants (86.7% [95% CI, 62.1%-96.3%]) compared with male participants (76.9% [95% CI, 49.7%-91.8%]) or in the older (≥60 years) population (100% [95% CI, 56.6%-100%]) compared with the younger (<60 years) population (78.3% [95% CI, 58.1–90.3%]) TABLE 2. Only 1 sample returned a negative result using the LumiraDx SARS-CoV-2 Ag Test: it had detectable levels of SARS-CoV-2 RNA within 31 PCR cycles using the TaqPath COVID-19 Combo Kit. All samples identified as SARS-CoV-2 positive within 30 cycles, as measured by the reference RT-PCR systems, were confirmed positive by the LumiraDx SARS-CoV-2 Ag Test, with a PPA of 100% (95% CI, 85.1%-100%; n = 22) TABLE 3. Variation between Ct values of the cobas 6800 SARS-CoV-2 Test and the TaqPath COVID-19 Combo Kit may have occurred owing to differences in gene targets. FIGURE 2 presents the results of the LumiraDx SARS-CoV-2 Ag Test by RT-PCR Ct values for each reference RT-PCR method and indicates the sensitivity of the test to detect antigen across the range of Ct values.

**TABLE 3:** LumiraDx SARS-CoV-2 Ag Test Performance in Cycle Threshold Subsets as Determined by the cobas 6800 SARS-CoV-2 Test (Roche Diagnostics), TaqPath COVID-19 Combo Kit (Thermo Fisher Scientific), and Either Reverse Transcription–Polymerase Chain Reaction Method

RT-PCR Test	Roche Diagnostics + Thermo Fisher Scientific		Roche Diagnostics		Thermo Fisher Scientific	
Ct Value	No.	PPA (95% CI), %	No.	PPA (95% CI), %	No.	PPA (95% CI), %
**<33**	24	95.8 (79.8-99.3)	8	100 (67.6-100)	16	93.8 (71.7-98.9)
**<30**	22	100 (85.1-100)	7	100 (64.6-100)	15	100 (79.6-100)
**<25**	18	100 (82.4-100)	6	100 (61.0-100)	12	100 (75.8-100)

CI, confidence interval; COVID-19, coronavirus disease 2019; Ct, cycle threshold; PPA; positive percent agreement; RT-PCR, reverse transcription–polymerase chain reaction; SARS-CoV-2, severe acute respiratory syndrome coronavirus 2.

## DISCUSSION

The LumiraDx SARS-CoV-2 Ag Test is the first rapid SARS-CoV-2 antigen test to demonstrate high agreement among asymptomatic individuals with COVID-19 compared with RT-PCR test results. The test performed well when the RT-PCR Ct was under 33 cycles, similar to results seen at Ct under 33 cycles for symptomatic participants.^20^

Many of the SARS-CoV-2 lateral flow rapid antigen tests currently available on the market have been shown to lack sensitivity and produce a high proportion of false-negative results, especially with asymptomatic participants, which can have a significant effect on containment of the COVID-19 pandemic when screening is required.^10-14^ In asymptomatic participants who were symptom-free for at least 2 weeks before testing, the LumiraDx SARS-CoV-2 Ag Test reported a PPA of 82.1% compared with reference RT-PCR systems in contrast to 97.6% previously reported for symptomatic participants within 12 days of symptom onset.^20^

The PPA was highest in samples in which SARS-CoV-2 RNA was detected within fewer than 33 cycles, with agreement of 95.8% at Ct under 33, and 100% PPA between the LumiraDx SARS-CoV-2 Ag Test and RT-PCR testing for all samples where RNA was detected within 30 RT-PCR cycles. Ct values correlate to the concentration of viral genetic material present in samples: a low Ct value represents high concentrations of viral RNA and vice versa.^26^ It has been proposed that individuals with SARS-CoV-2 RT-PCR Ct values exceeding 33 should not be considered an infection risk.^27^ Data obtained from asymptomatic participants in this study, together with data previously reported in symptomatic participants,^20^ indicate that the LumiraDx SARS-CoV-2 Ag Test detects samples to a Ct under 33 with high accuracy, regardless of whether the participant presents with symptoms. This finding is important because many antigen tests using lateral flow technology have limited sensitivity for antigen at high Ct values and in asymptomatic cohorts.^18^ Asymptomatic people present at various stages of infection: they have no means of identifying how long they have been infected or when they became infected because of the lack of symptoms and consequently may not be infective when tested.

A limitation of our study was the lack of medium-term follow-up of participants. It was therefore not possible to confirm whether participants who were asymptomatic at the time of testing later went on to develop symptoms or infect other individuals (with or without symptoms). They were, however, confirmed as symptom-free for 2 weeks before testing. Given the range of Ct values observed in the asymptomatic population screened, the analyses suggest that the LumiraDx SARS-CoV-2 Ag Test identified individuals, either asymptomatic or presymptomatic, with potentially the highest infection risk. Another limitation of the study was the small sample size of asymptomatic COVID-19–positive individuals identified during this study. Even though the sample size exceeds the minimal threshold set by the FDA, we acknowledge that limited conclusions can be drawn from stratification based on age, sex, and Ct values. That said, more than 200 participants were recruited, and asymptomatic individuals are difficult to identify because of the lack of symptoms. The age range of the participants and Ct range in the study are similar to those in other studies of antigen tests.^18,20^ Of note, 2 different RT-PCR assays were used in this study—namely, the cobas 6800 SARS-CoV-2 Test and the TaqPath COVID-19 Combo Kit. We acknowledge that variation between the 2 assays (resulting from differences in gene targets) may have led to a variation in Ct values and influenced the PPA values of the LumiraDx SARS-CoV-2 Ag Test. The Ct analysis in FIGURE 2, however, suggests that the results were similar between the 2 RT-PCR systems.

This study reported high agreement between the LumiraDx SARS-CoV-2 Ag Test and the reference RT-PCR test in asymptomatic participants who had been in recent contact with a COVID-19–positive individual or undergoing testing for work purposes. This study suggests that the microfluidic immunofluorescence POC LumiraDx SARS-CoV-2 Ag Test is a valuable tool for the rapid screening of individuals without symptoms in both community and health care settings to limit the spread of COVID-19.

## Notes

Funding: This work was supported by LumiraDx UK.
